# Coping Strategies to Enhance the Mental Wellbeing of Sexual and Gender Minority Youths: A Scoping Review

**DOI:** 10.3390/ijerph19148738

**Published:** 2022-07-18

**Authors:** Mathijs F. G. Lucassen, Alicia Núñez-García, Katharine A. Rimes, Louise M. Wallace, Katherine E. Brown, Rajvinder Samra

**Affiliations:** 1 Department of Health and Social Care, The Open University, Milton Keynes MK7 6AA, UK; alicia.nunez-garcia@open.ac.uk (A.N.-G.); louise.wallace@open.ac.uk (L.M.W.); rajvinder.samra@open.ac.uk (R.S.); 2 Department of Psychology, King’s College London, London SE5 8AF, UK; katharine.rimes@kcl.ac.uk; 3 Department of Psychology, Sport and Geography, University of Hertfordshire, Hatfield AL10 9AB, UK; k.brown25@herts.ac.uk

**Keywords:** LGBT, e-therapy, depression, adolescent, youth, online, sexuality, gender, resilience, scoping review

## Abstract

Robust population-based research has established that sexual and gender minority youths (SGMYs) are at an increased risk of mental ill-health, but there is a dearth of literature that seeks to explore how to best support SGMY mental wellbeing. This scoping review aims to identify findings related to coping strategies and/or interventions for building resilience and/or enhancing the mental wellbeing of SGMYs. PRISMA extension for scoping review (PRISMA-ScR) guidelines was utilized for this review. Studies were included if they were peer-reviewed papers containing primary data; reported psycho-social coping strategies for SGMY; were conducted with SGMYs in the adolescent age range; and were published in English. MEDLINE, Embase, and PsycINFO databases were searched. Of the 3692 papers initially identified, 68 papers were included with 24 intervention-focused studies of 17 unique interventions found. The most commonly cited therapeutic modality was cognitive behavioral therapy (CBT) (n = 11 studies). Despite the need to support the mental wellbeing of SGMYs, few interventions focused on this area and unique populations have been reported upon in the peer-reviewed literature. As a result, there is considerable potential to develop supports for SGMYs.

## 1. Introduction

Sexual and gender minority youths (SGMYs) include the young people who identify as lesbian, gay, bisexual, transgender/trans, and queer, as well as all other sexual and gender minority (i.e., LGBTQ+) youth. It is estimated that up to 10% of the adolescent population are SGMYs, based on the results of a range of population-based studies [1,2]. Prior research has indicated that SGMYs are more likely to experience mental health problems in comparison to their peers who are heterosexual and cisgender (i.e., not transgender). For example, a systematic review and meta-analysis of population-based studies reported sexual minority youths had three times the risk (odds ratio = 2.9) of depression in comparison to heterosexual youths [1]. Estimates of depression for gender minority youths indicate even greater mental health needs, with a nationally representative population-based estimate from New Zealand reporting gender minority youths had almost six times the odds (odds ratio = 5.7) of depression when compared to their peers who are cisgender [3].

The elevated rates of mental health problems frequently experienced by SGMYs are hypothesized to be largely driven by minority stress. The effects of the stigma, discrimination, or victimization that SGMYs can experience in their everyday lives, and the high and chronic levels of stress they face, place them at an increased risk [4,5]. In short, “…it is toxic social environments that place SGMY at elevated risk of mental ill-health” [6] (p. 6). Logically more needs to be done to improve these challenging social environments, over and above the work LGBTQ+ organizations have engaged with over many years to bring about positive change. Improving the overall milieu for SGMYs is therefore important, and some further efforts are underway to address these challenging environments, for example, the work associated with the UK’s “LGBT Action Plan 2018: Improving the lives of Lesbian, Gay, Bisexual and Transgender people” [7]. However, overall progress seems out of step with the relative size of the SGMY population, as well as the considerable mental health needs of SGMYs. For instance, there appears to be limited interventions specifically designed to support the mental health of SGMYs, as highlighted in three systematic reviews that sought to identify such resources [8,9,10].

Fortunately, considerable evidence is already available on how to treat commonly occurring mental health problems, such as for depression and anxiety in young people generally. For instance, the use of cognitive behavioral therapy (CBT) has been recommended for children and young people with mild through to severe depression in the UK’s influential National Institute for Health and Care Excellence/NICE treatment guidelines [11]. However, sexual and gender minority (SGM) people have not been well served by the mainstream health and social care services that are evidence-based [12,13,14]. For example, when Foy et al. [12] surveyed sexual minority adults about their treatment experiences, which frequently included CBT, half (52.2%) highlighted that services should be improved for sexual minority clients. Common issues identified by participants included their therapist’s lack of awareness and understanding of sexual minority identities and community-specific challenges, with distrust, disillusionment, and exclusion being common therapy experiences [12]. Furthermore, previous work has reported that the clinical outcomes from mainstream therapy services for SGM individuals are poor relative to those of their heterosexual cisgender peers [13,14]. This is especially the case for lesbian women and bisexual adults [13], as well as gender minority adolescents [14].

Given that mainstream services appear less acceptable and effective for SGM adults and gender minority youths, an investigation of the therapeutic potential and usefulness of supports for SGMYs more generally is warranted. However, internationally, only 24 such interventions have been identified and most of these (n = 17) have a sexual health focus [8]. SGMYs have highlighted a preference for digital psychosocial supports [15]. However, given the results of the reviews to date, few such digital interventions have been developed specifically to meet the needs of SGMYs [8,9,10]. We identified several broadly related literature reviews associated with the efforts to support SGMYs, but none had a focus on the specific coping strategies and/or interventions for building resilience and/or enhancing the mental wellbeing of SGMYs. For instance, there have been reviews with an emphasis on defining resilience, either for young people generally [16] or SGMYs more specifically [17]. Reviews have also described the SGMY inequities context [18] and have assisted our understanding of the overall methods that should be employed when working with SGMYs, including strength-based approaches [19]. However, it appears that there is scant evidence and explanations pertaining to the specific psycho-social skills or resources used within interventions that build resilience or promote mental wellbeing and coping strategies for SGMYs, either digitally or in-person. This gap in the knowledge-base is likely to go some way to explain the lack of tools being developed and offered for SGMY mental health promotion.

### 1.1. Rationale

As a study team, we are in the process of creating a bespoke digital intervention. This new resource is intended to build the resilience skills and enhance the mental wellbeing of SGMYs [6]. We have decided to conduct a scoping review to support us in developing this intervention for two key reasons. Firstly, because the digital intervention field is a fast-moving area, this time-efficient overarching review methodology is especially useful. Secondly, there is a gap in the literature in relation to what appears effective and acceptable for SGMYs in terms of evidence-informed psycho-social coping strategies and/or interventions.

### 1.2. Objective

Our objective is to identify the scope of evidence for the recommended psycho-social coping techniques or strategies for building resilience and/or enhancing the wellbeing of SGMYs in the adolescent age range.

## 2. Methods

### 2.1. Protocol and Registration

The reporting of this scoping review was guided by the Preferred Reporting Items for Systematic Reviews and Meta-Analyses extension for Scoping Reviews (PRISMA-ScR) checklist [20]. Given that we have time limited funding to design, co-create, and evaluate a new web-based intervention (by the end of 2022), this scoping review was not registered prior to being conducted.

### 2.2. Eligibility Criteria

The eligibility criteria for our scoping review, from database inception to January 2022, were as follows. Publications were included if they were:

- Peer-reviewed papers containing primary data (using quantitative, qualitative, or mixed-methods research designs);
- Papers that reported on what is considered effective or useful in terms of psycho-social coping strategies for SGMYs;
- Studies that were conducted with SGMYs in the adolescent age range, which could potentially include participants as young as 10 years and as old as 19 years (i.e., the World Health Organization/WHO definition of adolescence), or where the sample included adults (or children) then more than 50% of the study’s participants are adolescents;
- Were published in English.
- Publications were excluded if they were:
- Studies where adolescent data were not presented separately from adult or child data;
- Literature reviews;
- Opinion pieces, commentaries, or theoretical pieces (i.e., the publication did not contain original data);
- Conference abstracts (i.e., only a brief summary of the research conducted);
- Dissertations.

We excluded papers that did not focus on adolescents as we aimed to create a developmentally appropriate resource for SGMYs. Moreover, we only included original data from peer-reviewed papers, as we are interested in summaries of the empirical research and what has been concluded based on that data.

### 2.3. Information Sources and Search Details

Three databases were searched, specifically MEDLINE, Embase, and PsycInfo from their inception to 19 January 2022. The searches across the three databases were based on the search terms used for MEDLINE. The search strategy for MEDLINE was built by three of the authors (i.e., M.F.G.L., R.S., and K.A.R.) and was then reviewed and refined by the remaining authors (i.e., A.N.-G., L.M.W., and K.E.B.) before the full search was conducted. For MEDLINE, the searching was from the database’s inception (which was in 1946) to 19 January 2022 (for the specific search terms used, see Appendix A). The same electronic limits were applied to all three databases, specifically that all papers were to be published in the English language, involved humans, and had adolescent participants.

### 2.4. Selection of Evidence and Data Processes

M.F.G.L. screened all the titles and abstracts from the papers initially identified. The full-text papers were all checked by both M.F.G.L. and A.N.-G. and any disagreements about their inclusion were resolved via discussion with R.S. and K.A.R. Nine articles were identified by checking the reference lists of the included studies. Where it was not clear whether or not a paper should have been included, due to a lack of demographic information in the actual paper, the corresponding author was contacted. All data extraction and reporting were conducted by M.F.G.L. and independently checked by A.N.-G. Key information collated included details about the author(s); year of publication; study location; the sample size; how the target population was defined; the salient features of psycho-social coping techniques or strategies for SGMYs identified; and (where applicable) the format of the intervention. Given the diverse ways in which SGMY populations are described, the language as utilized in the individual papers was employed when the paper was summarized.

### 2.5. Appraisal of Individual Sources of Evidence and Synthesis of Results

Prior to commencing this work, we initially debated focusing our review exclusively on trials of mental health interventions conducted with SGMYs. However, it was apparent during the early stages of developing our search terms that such a restrictive focus would be counter-productive, due to the small number of trials identified in the reviews already conducted. A conventional critical appraisal of the quality of the evidence was outside the scope of this review, which was designed to identify the key psychosocial techniques that have been employed for and by SGMYs. Consequently, we have taken a more descriptive approach, as recommended by Arksey and O’Malley [21], where we have not rated the evidence per se, but have outlined the common focus, features, and format of interventions; the standardized assessments used; and the significant clinical or other outcomes reported. We also sought to summarize the expert by experience insights into potentially effective strategies that were provided by SGMYs, as outlined in a range of qualitative studies. Arksey and O’Malley’s [21] framework informed the overall process and synthesis of the review, using their five stages of identifying the research question; identifying relevant studies; study selection; charting the data; and collating, summarizing, and reporting the results.

## 3. Results

### 3.1. Selection of Sources of Evidence

The initial results yielded a total of 3692 articles that were from:

- MEDLINE = 1100 articles;
- Embase = 1226 articles;
- PsycInfo = 1366 articles.

**Figure 1:**
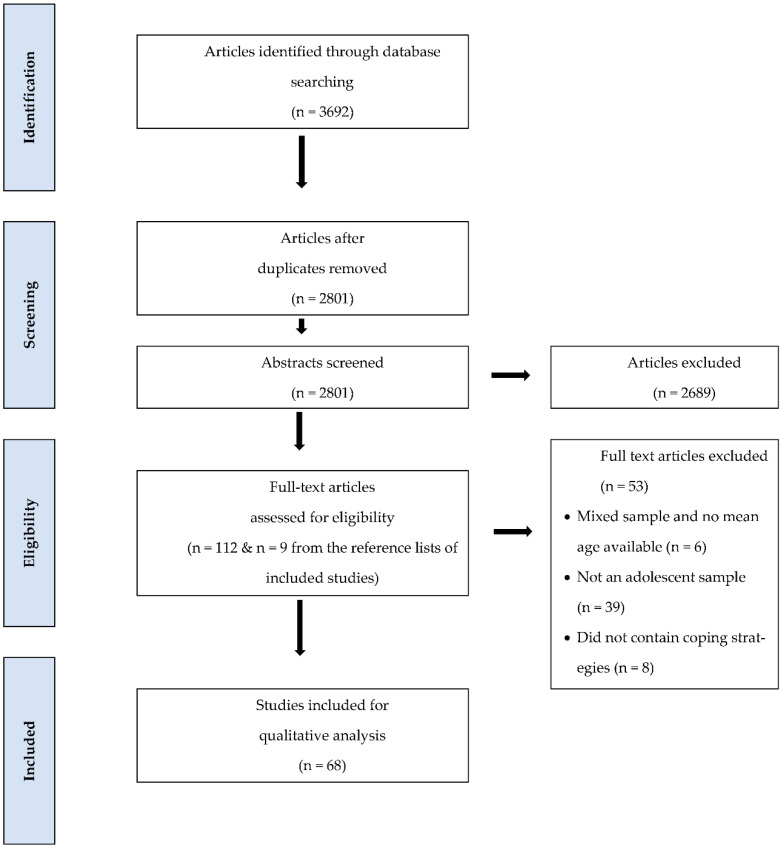
PRISMA flow diagram for scoping review.

Once the duplicates were removed, there were 2801 articles identified for title and abstract screening. Of these articles, 2689 were excluded and 112 were identified for a full-text review, 59 of these full-texts were included in this review and an additional 9 papers were identified after reviewing the reference lists of the included full texts. As a result, 68 papers were included in our review (see Figure 1 for details).

### 3.2. Overall Characteristics of the Sources of Evidence

In total, 68 studies were included in this review. The research in the field is fairly recent, as the oldest papers date from 2008 and more than half of the studies have been published from 2017 onwards (n = 35 studies). Most studies involved fewer than 50 participants (n = 40 studies). North American research predominates, with over two-thirds of papers consisting of participants from the USA (n = 47), including five studies where the participants were from both Canada and the USA. Other studies were conducted in a single country, specifically Canada (n = 5), the United Kingdom (n = 4), New Zealand (n = 4), and Australia (n = 2), with one study each conducted in South Africa, Belgium, Puerto Rico, Israel, and Norway. One study drew participants from across ten African countries (i.e., [22]). There was no uniform focus on the LGBTQ+ sub-populations of interest, but the studies could be broadly categorized into one of three main groups:

1. Those that consisted of LGB (or sexual minority) youths (n = 26 studies).
2. Those that consisted of LGBTQ/+ (or sexual and gender minority) youths (n = 28 studies).
3. Those that consisted of transgender/trans (or gender minority) youths (n = 14 studies).

The majority of studies (n = 41, 60.3%) provided details of the funder/s of the research (e.g., government ministries, individual universities, and research funding councils); the remainder did not explicitly acknowledge a funder or funders.

### 3.3. Appraisal of the Intervention-Focused Studies (n = 24)

Of the 68 papers, 24 were intervention-focused. Of these, more than half (n = 13 studies) included SGMY/LGBTQ+ youths and the remainder either focused on sexual minority/LGB young people (n = 9 studies) or gender minority/trans young people (n = 2 studies). Table 1 provides an overview of these 24 studies (which represent 17 unique interventions). Of the 17 unique interventions, most were primarily psychotherapeutic in nature (n = 12). A smaller number were, broadly speaking, preventive or universal interventions (n = 5), which were designed either for SGMYs specifically or for all young people more generally.

The majority of studies were focused on the initial development or assessment of novel interventions for SGMYs. The most commonly cited therapeutic modality was cognitive behavioral therapy (n = 11 studies), which was utilized in “AFFIRM” (n = 4 studies), “SPARX/Rainbow SPARX” (n = 3 studies), “ASSET” (n = 1 study), and in unnamed interventions for three studies (i.e., [23,24,25]). Two interventions consisted of family therapy, specifically “Familias con Orgullo/Families with Pride”, and an unnamed intervention (i.e., [26]).

Most of the interventions were provided in-person (n = 14 studies) in either individual or group formats, with four being delivered in schools (i.e., [24,27,28,29]). Five interventions (n = 9 studies) were provided digitally, in synchronous (e.g., for “Q-Chat Space”) or asynchronous formats (e.g., for “Singularities”). One study explored the potential of a yet-to-be-created intervention, and, as such, it was yet to be determined whether the eventual intervention would be delivered digitally (i.e., [30]). Two of the interventions delivered digitally (n = 5 studies) were provided in serious game formats (i.e., “Singularities” and “SPARX/Rainbow SPARX”).

Most commonly, the study designs were pilot studies (n = 9) and only one was a randomized controlled trial (i.e., [31]). The focus of the pilot studies was predominantly testing the acceptability, feasibility, and/or preliminary effectiveness of the interventions. A range of standardized assessments were used across the pilot studies, such as measures of depressive symptoms (e.g., the Beck Depression Inventory). Given the design of these studies (i.e., small-scale open trials/pilots), it is unknown if any of the interventions are effective, although acceptability data appear positive based on the feedback from SGMYs.

### 3.4. Summary of the Interventions’ Content

Table 2 provides an overview of the therapeutic content or coping strategies that were common across the interventions. These could be broadly categorized as cognitive/emotional or cognitive (n = 6), environmental/social (n = 5), or behavioral (n = 2). Many were evidence-based techniques adapted for SGMYs. For instance, CBT techniques, such as cognitive restructuring, where the “ABCD” cognitive restructuring method (as outlined in “AFFIRM”) was applied, in particular, where this method was then linked to salient experiences with accompanying suggested responses for SGMYs, such as in this example:

“A [Activating event]: I am genderqueer. B [Belief or thought]: “No one can be happy if they are genderqueer,” or “Being genderqueer is going to ruin my life,” and “I won’t be able to handle the discrimination and stigma associated with being genderqueer.” C [Consequence of your thought]: I feel hopeless and worried. D [Dispute or talk back to your thought]: “There are people who are genderqueer who are as happy as people with other identities.” “Discrimination against genderqueer people happens and it is awful, but it won’t ruin each minute of my life.” “I am a strong and determined person, who can have a good life in spite of discrimination.” “Instead of wasting energy doubting myself and feeling anxiety, I can use my energy to figure out the best way to live an authentic life”

[33] (p. 5).

Of note, all the interventions were affirming of SGMYs’ identities. Although not a technique per se, the instillation of hope for SGMYs was recognized as important, for instance, in “AFFIRM” [34] and “Rainbow SPARX” [42]. Where the latter stated to users of the program:

“The other message in the game [Rainbow SPARX] was about having hope. It’s good to repeat these simple messages: “I won’t always feel this way”; “Things will get better”; or “It can be hard not being straight, but I know I can handle the challenges that come my way.” “These statements are true and thinking them can make you feel a little better almost instantly, even if you don’t believe them at first”

[42] (p. 206).

### 3.5. Appraisal of the Non-Intervention-Focused Studies (n = 44)

Table 3 summarizes the methods and key findings obtained from the non-intervention-focused studies, which frequently drew upon the expert by experience perspectives of SGMYs in regard to psycho-social coping techniques or strategies. Repeatedly, in the included studies, the ability to obtain support and a connection with other SGMYs was seen as important (e.g., [46,47,48,49,50,51]). The opportunity to meet people “like me” was seen as especially useful. This point was reinforced by a participant who highlighted: “…being surrounded by so many LGBTQ community members and allies convinced me that I can one day feel as happy, safe, and loved all the time” [48] (p. 55).

The Internet was frequently seen as an important way in which SGMYs could achieve a connection with other SGMYs for support purposes (e.g., [52,53,54,55,56,57,58]). The Internet was even described as “life saving” for SGMYs [59]. This point was reinforced by an SGMY who stated: “There’s a supportive community out there online and they mean the world to me– they’ve saved my life” [59] (p. 37). However, the Internet could also be problematic for SGMYs [56,60]. For example, SGMYs could be exposed to mistreatment online, with one adolescent noting: “It’s [social media] public. Which is both a blessing and a curse because you can connect with all these people but also you are open to a lot of hate” [56] (p. 278). Even social media groups, specifically for SGMYs, could be a source of discrimination and stigma [55]. For instance, certain SGMYs expressed racist or transphobic views or engaged in exclusionary behavior within a group, expressing sentiments such as: “you cannot be in here, you are not gay enough’’ [55] (p. 426). As a result of online issues, SGMYs are required to be skillful users of the Internet, such as when they use certain platform features to protect themselves (e.g., by utilizing blocking and privacy settings for safety reasons) [60].

SGMYs taking on an educator role (i.e., [56,61]) or something akin to a political advocate role (i.e., [46,57,62]) was perceived as helpful by SGMYs. For instance, when SGMYs held a “proud LGBTQ+ position”, this could be resilience-enhancing in the face of mistreatment [62]. Engaging in altruistic activities or roles where SGMYs were “giving back” was also thought to be beneficial (i.e., [59,60,63]). This included SGMYs mentoring other SGMYs (i.e., [63,64]) or providing online support to SGMYs [60]—which in turn helped them “feel good after helping their peers” [60] (p. 171).

SGMYs also sought to “escape” from challenging environments (i.e., [51,59,65]) and they created “pockets of safety” [61] for themselves. They used cognitive strategies to manage negative messages, such as those of a religious nature [52,61,66]. An example is when an SGMY reflected: “Because I believe God made everybody, so if God didn’t want people to be gay, then God wouldn’t have made them gay” [52] (p. 7).

Being “out” in terms of one’s sexuality and/or gender identity proffered both potential wellbeing benefits and challenges. In some instances, “learning to hide” [67], as it was described in South Africa, or being “being closeted”, as cited in Belgium [68], could be adaptive given the potential negative reactions of others towards “out” SGMYs. This meant that SGMYs used “…a closed visibility management strategy in specific social situations that are perceived as risky” [68] (p. 697). A similar point was also made by Rubin and McClelland [69] in their research on queer American women of color, where concerns about possible homophobic comments from peers meant that deleting their social media profile was an adaptive way to maintain personal safety. By contrast, being out has the potential to increase the amount of support that SGMYs receive (i.e., [61]). This can be the case, despite the “many cultural and familial taboos” SGMYs can experience [61] (p. 628).

Certain behavioral techniques were described as being valuable in terms of supporting mental wellbeing, including diversionary activities (i.e., [70,71]). For example, Strauss and colleagues [70] noted that amongst trans and gender diverse young people, being distracted by social media, games, or watching online media “…took their minds off their concerns, at least momentarily” [70] (p. 5). Physical exercise was also cited as important (i.e., [71,72]), especially during the COVID-19 pandemic where exercise and outdoor activities provided benefits to mental wellbeing. As noted by an SGMY, “…the lack of sports has really had a negative impact on me [during the pandemic]. It’s hard to motivate myself, but I always feel better after exercising” [72] (p. 1055). These activities were not merely distractions, but could also provide a way to feel more positive about their identity [71], as demonstrated by an SGMY in relation to going to the gym as a coping strategy. He did this when confronted with anti-LGBTQ+ experiences, stating: “I feel the frustration, the anger—it’s like the fuel for me to work out and push harder. It kinda turns into a positive. I feel better.” [71] (p. 145).

SGMYs can engage in coping strategies usually viewed by others as problematic, in particular, self-harming (i.e., [60,73,74]). However, for some LGBTQ+ participants, their self-harming was perceived to be a positive coping strategy [75]. Other coping strategies that caused harm and/or serious risk to the young person included suicide attempts, risky sexual practices, and excessive drinking and recreational drug-taking [22,62,74].

Certain psychological strategies were also described. For example, in terms of coping with victimization, participants described using mindfulness and emotional regulation strategies; cognitive reappraisals; assertive communication techniques; and questioning and resisting rigid culturally bound labels [57,71,76]. Some used apathy as a response to emotional pain (i.e., [52,77]). Avoidance—both psychologically and physically—was also a perceived coping strategy (i.e., [50,60]). Regarding emotional avoidance, participants suppressed emotions as a means to block these challenging feelings, in an attempt to avoid pain and humiliation [67]. Ignoring or avoiding certain people or behavior was a strategy utilized to reduce the likelihood of distress, and hence preserve emotional energy [78]. For instance, as highlighted by an SGMY: “…I know when to just drop it and walk away/block them when they aren’t open to learning. Negative comments online are inevitable and they can be hurtful… You have the opportunity to teach them but if they aren’t open minded, you can simply ignore them…” [60] (p. 170).

## 4. Discussion

The issues SGMYs face, from physical violence to unsupportive families, have been well documented in the literature, but there is much less research on how SGMYs can best cope with environments that are often hostile to them. The present scoping review supports the earlier findings highlighting the relative paucity of interventions designed to support the mental wellbeing of SGMYs, including those that are web-based in format [8,9,10]. However, a UK Department of Health commissioned report highlighted SGMYs’ strong preference to access help on the Internet, whereby 82.3% (n = 572) of SGMY participants reported that they would be “likely” or “very likely” to select help in this format [89]. Prior reviews have not delved into the therapeutic content of interventions, or the specific strategies designed to be effective in supporting the mental wellbeing of SGMYs, which represents a key strength of the present review.

Our current review indicates that a range of techniques or coping strategies have been considered as effective across cognitive or cognitive/emotional, environmental/social, and behavioral domains. When incorporated within interventions, the majority have been provided in-person. Therefore, there exists an untapped potential in adapting successful psychotherapeutic techniques to a Web-based format, in line with SGMYs’ preferences. In relation to the intervention-focused studies, many of the techniques or strategies were evidence-informed and adapted to meet the needs of SGMYs. For example, the CBT techniques of cognitive restructuring and behavioral activation, adapted to be usefully applied to the lives of SGMYs, are adaptations that have long been advocated by psychotherapy experts in the field of SGMY mental health (i.e., [90,91]).

The review findings suggest some challenges for the developers of Web-based interventions for SGMYs. For instance, the ability for SGMYs to connect with other SGMYs has been reinforced as particularly important in regard to supporting the mental wellbeing of these young people. However, this connecting requires careful considerations if provided online, given the feedback provided earlier by both professionals and SGMYs about the risks [15]. Specifically, there are risks concerning online stranger connections, and Internet safety and security issues, including the risk of SGMYs being outed on the Web or specifically targeted for sexual exploitation. There are also serious challenges associated with how suicidality is safely managed in a Web-based context [15]. Another salient challenge is ensuring that interventions do not neglect the needs of SGMYs with other socially disadvantaged characteristics, such as minority race/ethnicity, disability, or female sex. Therefore, future interventions should represent multiple identities by fully acknowledging the intersections between SGM status [65], and other minority characteristics. In part, this could be achieved by using authentic portrayals of LGBTQ+ people, as suggested by Davis and colleagues [80], which would ensure that people with multiple identities are included.

Many simple strategies should be straightforward to embed in future Web-based and other interventions, such as psychoeducation pertaining to minority stress and its impact on SGMYs, as well as basic behavioral techniques, such as relaxations exercises. Youth-friendly and inclusive language adopted in interventions should also be easy to apply and readily adopted in future Web-based interventions, specifically the promotion of inclusivity by using an SGMY’s preferred name and pronouns to model transgender supportiveness in resources. Interventions should also strive to use developmentally appropriate language, so that the terminology employed is easily understood by younger SGMYs. Therefore, therapy jargon and psychological concepts will need to be outlined suitably and acceptably to SGMYs.

### Strengths and Limitations

To the best of our knowledge, the present review is the first to identify the recommended psycho-social coping techniques or strategies for building resilience and/or enhancing the wellbeing of SGMYs, based on the primary data published in peer-reviewed papers. A drawback of this approach is that we did not explore the gray literature and could have potentially missed relevant findings as a result. Our analysis of the included studies consisted of a narrative synthesis, as outlined by Arksey and O’Malley [21], which was required in this review due to it focusing on investigating the types of psychosocial techniques reported in the previous research, rather than quantifying the quality of the included studies or their success. In particular, given the diverse nature of the research reported upon, which included quantitative, qualitative, and mixed-method studies, a conventional sensitivity analysis (similar to those frequently applied to quantitative studies) was not possible. Moreover, given the heterogenous nature of the existing literature and the lack of intervention trials, a critical appraisal of the clinical outcomes associated with the intervention-focused studies is currently premature. As is commonplace, we only included papers published in the English language, due to our limited resources [92]. That this scoping review only included studies published in English means that the potential insights from papers written in all other languages will have been missed. Studies from a range of countries were included, such as research conducted in South Africa, Israel, and Puerto Rico. However, over two-thirds of the studies were from a single high-income Western nation, specifically the USA. American predominance in psycho-social research is not new [93], but this focus on the USA does create a bias we would like to acknowledge. Of relevance to the research on LGBTQ+ people is that studies will likely be absent in countries where social environments make basic survival challenging for SGMYs, such as in the countries where there is a death penalty for “homosexual acts”. By contrast, across a range of more progressive countries, there has been considerable social progress for LGBTQ+ individuals (e.g., marriage equality). However, many SGMYs continue to face challenging social environments, which need to be improved. Whilst the focus of this review was on the coping strategies SGMYs could employ themselves, subsequent work should explore what is likely to be effective in regard to improving social environments for SGMYs. Fortunately, related work is underway, such as a PROSPERO registered review entitled “A realist evidence synthesis of mechanisms by which school-based interventions may widen or reduce inequalities in LGBT adolescents’ mental health”. We set our inclusion and exclusion criteria to emphasize an adolescent sample of SGMYs and we were holistic in terms of what was to be included regarding a study’s design and particular focus. Others may have decided to make different decisions in shaping a comparable review; nevertheless, we strove to be transparent and have provided details around our searching of the literature and our rationale.

## 5. Conclusions

For years now, the unique issues and mental health challenges that SGMYs commonly face have been evidenced in considerable depth in the research literature. It is now timely to start developing robust and evidence-informed interventions that seek to assist SGMYs best manage their adolescent years, which are often experienced in unsupportive environments. This review provides an overview of the coping strategies designed to enhance the mental wellbeing of SGMYs, and, as such, it can be used to support efforts in assisting SGMYs to thrive. A range of strategies and interventions appear promising for use in Web-based tools, in order to support the mental wellbeing of SGMYs. However, issues around appropriately managing peer-to-peer online interactions and inclusion, especially for youth who experience multiple social disadvantages, will require careful consideration.

## Figures and Tables

**Table 1:** Intervention-focused studies (n = 24 studies).

Intervention (Modality/Focus) *In-Person or Digital* [Psychotherapeutic or Preventive/Universal]	Author/s (Year) Country	Study Design	Focus of the Intervention/Study	Sample ^a^ (Number) (Age Range) [Mean Age] ^b^	Intervention Description	Standardized Measures Used	Main Clinical or Outcome Results
“AFFIRM” (CBT) *In-person* [Psychotherapeutic]	Austin and Craig (2015) [32] USA	Qualitative study using focus groups and interviews	Developing an affirmative version of CBT for SGMYs	Sexual and gender minority youths (n = 28) (High-school aged) [≤19 years]	AFFIRM is an 8-module affirmative CBT group intervention developed for LGBTQ+ youths. Targets identity-based stressors (e.g., transphobic bullying) that contribute to emotional distress and seeks to improve functioning by addressing underlying, problematic cognitions. It explores how SGMYs have learned to cope with identity-specific stressors, facilitates the development of affirming and “realistic alternative ways of thinking and behaving” (p. 138), and enhances connection to and support from others.	No standardized assessments	Not applicable
	Austin, Craig, and D’Souza (2018) [33] Canada	Pilot study (open trial)	Preliminary effectiveness and acceptability of AFFIRM	Transgender youths (n = 8) (16–18 years) [17.6 years]		Beck Depression Inventory, a Reflective Coping Subscale, and an AFFIRM satisfaction survey	Significant reductions in depression scores, non-significant changes in coping, and 7/8 (87.5%) participants would recommend AFFIRM to other SGMYs.
	Craig and Austin (2016) [34] Canada	Pilot study (open trial)	Feasibility, acceptability, and effectiveness of AFFIRM	Sexual and gender minority youths (n = 30) (15–18 years) [17.1 years]		Beck Depression Inventory, Stress Appraisal Measure for Adolescents, a Reflective Coping Subscale, and a satisfaction survey	Significant reductions in depression scores, reflective coping and stress appraisal reported, and 97% would recommend AFFIRM to others. Attendance and treatment completion was 100%.
	Craig et al., (2018) [35] Canada	Pilot study follow-up from Craig and Austin (2016)	Pre- to post-intervention changes in the coping strategies of AFFIRM participants	Sexual and gender minority youths (n = 30) (15–18 years) [17.1 years]		Adolescent Coping Orientation for Problem Experiences	Significant increase in use of engagement coping (e.g., being humorous and seeking spiritual support), as well as primary control (solving family issues).
“Singularities” (a theory-based, community-informed game) *Digital* [Psychotherapeutic]	Coulter et al., (2019) [36] USA	Study protocol for 2 arm RCT (with some preliminary demo-graphic data of those recruited)	Development of intervention and protocol as well as feasibility of design for randomized controlled trial	Sexual and gender minority youths (n = 240) (14–18 years) [≤19 years]	A serious game that encourages help-seeking and productive coping and raises awareness of online resources. For every nonplayable character/NPC that is successfully helped, the participant gets a positive story ending.	Multiple assessments, e.g., an adapted version of the Cyberbullying Perpetration Scale and the Patient Health Questionnaire-9 (for children)	Not applicable
	Egan et al., (2021) [31] USA	RCT [n = 120 in the control condition (a list of resources)]	Acceptability, feasibility, and preliminary effectiveness	Sexual and gender minority youths (n = 240) (14–18 years) [≤19 years]			Significant reductions in cyberbullying victimization, binge drinking, and marijuana use frequency. Over half downloaded the game (55.8%) and of the players 50.8% would recommend it.
“ASSET” (CBT) *In-person* [Psychotherapeutic]	Craig et al., (2014) [27] Canada	Pilot study (open trial)	Preliminary effectiveness and acceptability of ASSET	Sexual minority youths (n = 263) (13–20 years) [16.7 years]	Between 8–10 group sessions for SMYs that “…promoted effective problem solving and proactive coping skills…” (p. 92).	Rosenberg Self-Esteem Scale, Proactive Coping Inventory, Social Connectedness Scale, and a satisfaction survey	Significant increases in self-esteem and proactive coping—and the results were consistent across sub-groups (e.g., across race/ethnicity and gender). Low dropout (11%) and mean score of 3.8 (maximum 4) for “I would recommend this program to other LGBTQ youth”.
An unnamed intervention (CBT) *In-person* [Psychotherapeutic]	Craig et al., (2013) [25] USA	Case study (refined CBT for SGMYs)	Treatment of depression—and feelings of guilt and hopelessness	A bisexual female Hispanic adolescent (n = 1) (16 years old)	Adapted CBT—e.g., with cognitive restructuring, question the helpfulness (as opposed to the validity) of the thought or belief, and build skills for interacting within challenging environments.	No standardized assessments	Participant was provided with sources of potential social support (e.g., a gay–straight alliance)—no clinical outcomes reported.
An attachment-based intervention (family therapy) *In-person* [Psychotherapeutic]	Diamond et al., (2013) [26] USA	Pilot study (open trial)	Preliminary effectiveness and feasibility	LGB adolescents (n = 10) (14–18 years) [15.1 years] and their parents	Early focus in treatment on promoting adolescents’ access to, and participation in, LGB affirmative supports was important. A key goal was to increase awareness of and reduce the frequency of “…subtle yet subversive invalidating parental responses…” (p. 94).	Suicidal Ideation Questionnaire, Beck Depression Inventory, and Relationship Structures Questionnaire	Significant reductions in suicidal ideation, depression scores, and maternal attachment-related anxiety and avoidance. 8 out of 10 adolescents and their families completed treatment (on average 12 sessions each).
A culturally adapted intervention (CBT) *In-person* [Psychotherapeutic]	Duarté-Vélez at al., (2010) [23] Puerto Rico	Case study	Treatment of major depressive disorder	A gay male Latino adolescent (n = 1) (16 years old)	Addressed certain cognitions related to areas of conflict (i.e., sexuality, family, and spirituality) as these produced distress. Behavioral work focused on increasing pleasant activities (i.e., dancing) even if unacceptable to his family as “that is the work of homosexuals” (p. 902).	Multiple assessments, e.g., Children’s Depression Rating Scale-Revised, Dysfunctional Attitudes Scale, and Children’s Depression Inventory	Post-intervention the participant no longer met criteria for major depressive disorder.
“Q-Chat Space” (a chat-based support program) *Digital* [Psychotherapeutic]	Fish et al., (2020) [37] USA	Secondary analysis (of session transcripts)	Exploring the impact of the COVID-19 pandemic on LGBTQ youths	LGBTQ youths (n = 159) (13–19 years) [≤19 years]	Adult-facilitated text-based group intervention—“Youth often engaged in strategies to build rapport, foster community, and support each other” (p. 134). They sought advice, provided mutual validation, and recommended resources.	No standardized assessments	Not applicable
	Fish et al., (2021) [38] USA	Pilot study (open trial)	Utility, feasibility, and acceptability	LGBTQ youths (n = 236) of which n = 176 were users (13–19 years) [16.2 years]		An adapted assessment from the Family Acceptance Project and Kessler 6	Non-significant differences between users and non-users in terms of psychological distress. >1000 groups delivered overall. High levels of satisfaction with facilitators and chat topics (average > 4, 5 = maximum).
“Brave Trails” (a summer camp intervention) *In-person* [Preventive/universal]	Gillig, Miller, and Cox (2019) [39] USA	Pilot study (open trial)	Preliminary effectiveness	LGBTQ youths (n = 56) (12–20 years) [15.4 years]	This summer camp intervention includes free-choice programs (e.g., hiking with a counselor), workshops (e.g., “Self-Love 101” p. 371), build-on programs (e.g., writing a skit), and Brave Trails’ social entrepreneurship course (i.e., articulating a “story of self” for use to promote social change or advocacy).	Multiple assessments, e.g., adapted versions of the Center for Epidemiologic Studies Depression Scale and Resilience Scale	Significant increases in identity affirmation, hope and resilience, as well as a significant reduction in depression scores.
Unnamed intervention (to support coming out) *Digital N/A* [Preventive/universal]	Grafsky and Gary (2018) [30] USA	Qualitative study using interviews and open-ended surveys	Determine what would be most useful in a coming-out program	Sexual minority youths (n = 48) (14–22 years) [19.0 years]	Five themes—“Program Structure”, “Program Facilitator”, “Support”, “Education”, and “Sharing Stories” (e.g., hearing stories from others or sharing their own). Value of connecting with other SMYs reinforced.	No standardized assessments	Not applicable
An evaluation of LGBT- related school interventions/resources (education interventions) *In-person* [Preventive/universal]	Greytak et al., (2013) [29] USA	Cross-sectional survey	Preliminary effectiveness of education resources/interventions	LGBT (n = 6853) (13–21 years) [16.3 years]	Four interventions/resources: gay–straight alliances; supportive educators; LGBT-inclusive curricula; and comprehensive anti-LGBT bullying/harassment policies.	No standardized assessments	Three of the four interventions/resources (except for comprehensive anti-bullying/harassment policies) were associated with lower levels of victimization.
A mental health promotion program (CBT) *In-person* [Psychotherapeutic]	Heck (2015) [24] USA	Pilot study (open trial)	Feasibility and acceptability	LGBTQ (n = 10) (15–19 years 10th–12th grade) [≤19 years]	After a focus on identifying minority and general stressors, sessions emphasized developing cognitive coping, affect regulation, and problem-solving skills.	No standardized assessments	The mean number of sessions attended (of the maximum 4 sessions) was 2.4. “I think this session would be helpful for LGBTQ youth” was rated between 3.17 to 3.83 for the sessions (4 = maximum).
“Familias con Orgullo” (family therapy) *Digital* [Psychotherapeutic]	Lozano et al., (2021) [40] USA	Qualitative study evaluating therapy using interviews and focus groups	To describe the user-centered development of the intervention	Latinx sexual minority youths (n = 12) (13–17 years) [≤19 years] and parents	Adolescent-only content of the intervention focused on enhancing communication and supportive relationships, building empowerment and resilience, and addressing adolescent sexual health. Latinx cultural content highlighted as necessary.	No standardized assessments	Not applicable
“Rainbow YOUTH workshops” (an education intervention) *In-person* [Preventive/universal]	Lucassen and Burford (2015) [28] New Zealand	A mixed methods open trial	Preliminary effectiveness and acceptability	Sexual minority youths/SMY focus (% SMYs not established) (n = 229) (12–15 years) [13.7 years]	Intervention designed to improve school environments. Content included a “storyteller” discussing their “coming out” and “…what they found supportive during hard times….” (p. 546).	No standardized assessments	89.1% completed the both the pre- and post-workshop questionnaires (i.e., attended the whole workshop). 90.9% would recommend the workshop to other young people.
“SPARX” and “Rainbow SPARX” (CBT) *Digital* [Psychotherapeutic]	Lucassen et al., (2015) [41] New Zealand	Qualitative study evaluating Rainbow SPARX using interviews	Acceptability and perceived usefulness	Sexual minority youths (n = 25) (13–19 years) [16.4 years]	SPARX for SMY—included strengths-based views, e.g., “…It can be hard not being straight, but I know I can handle the challenges that come my way”, then “These statements are true and thinking them can make you feel a little better almost instantly, even if you do not believe them at first” (p. 206).	No standardized assessments	Participants identified appealing aspects (as well as “things to improve”) and 17/25 participants thought the intervention helped them feel better/less depressed.
	Lucassen et al., (2015) [42] New Zealand	Pilot study (open trial)	Acceptability, feasibility, and preliminary effectiveness	Sexual minority youths (n = 21) (13–19 years) [16.5 years]		Multiple assessments, e.g., Children’s Depression Rating Scale–Revised and the Spence Children’s Anxiety Scale	Significant reduction in depression, anxiety, and hopelessness scores. 91% completed intervention and 80% would recommend the intervention to friends.
	Lucassen et al., (2021) [14] New Zealand	Open trial	“Real world” assessment of SPARX	Transgender adolescents (n = 207) [and n = 2904 males and n = 5968 females] (12–19 years) [≤19 years]	Content included relaxation training, “do it” (e.g., behavioral activation), “sort it” (e.g., social skills training), “spot it” (recognize or name cognitive distortions), “solve it” (problem solving content), and “swap it” (e.g., cognitive restructuring).	Patient Health Questionnaire-modified for Adolescents	Male and female cisgender registrants had significant improvements in their scores, whereas transgender adolescents did not.
“Project YES” (single session interventions) *Digital* [Psychotherapeutic]	McDanal et al., (2022) [43] USA	A pre- to post- therapy mixed-methods evaluation	Acceptability and preliminary effectiveness	LGBTQ+ (n = 156) [and n = 102 heterosexual and cisgender youths] (11–17 years) [≤19 years]	“Project Personality” focuses on the malleability of traits/symptoms in order to strengthen perceived control and reduce hopelessness, “Project CARE” focuses on acting with self-compassion to reduce self-hate, and “Project ABC” focuses on behavioral activation principles to improve mood.	State Hope Scale, Beck Hopelessness Scale-4, Self-Hate Scale, and a program feedback scale/survey	Significant reductions in hopelessness and self-hate for cisgender LGBQ+, trans and gender diverse, and cisgender heterosexual youths. Values of >3 on the intervention feedback scale (5 = maximum).
“CMHI” (face-to-face services and supports) *In-person* [Psychotherapeutic]	Painter et al., (2018) [44] USA	Secondary analysis of data (service user data)	Evaluation of functional outcomes	LGBTQ (n = 482) [and n = 2726 heterosexual and cisgender youths] (11–21 years) [≤19 years]	The Comprehensive Community Mental Health Services for Children with Serious Emotional Disturbances Program/“CMHI” consisted of individual therapy, medication treatment, and case management.	Multiple assessments, e.g., The Youth Information Questionnaire Revised and the Child Behavior Checklist 6–18	Significant improvements reported for anxiety and depression for LGBTQ youths.
“Hatch Youth” (a group-based intervention) *In-person* [Preventive/universal]	Wilkerson et al., (2017) [45] USA	Evaluation of sessions using a cross-sectional survey	Indications of possible effectiveness	LGBTQ (n = 108) (13–20 years) [16.8 years]	Meetings arranged into three 1 h sections, specifically: unstructured social time; consciousness-raising (e.g., a presentation on the history of LGBTQ+ oppression); and a youth-led peer support group.	Multiple assessments, e.g., items from the Center for Epidemiological Studies Depression Scale	Those attending for 1–6 or ˃6 months reported higher social support, which was associated with improvements (e.g., decreased depression scores).

^a^ LGBTQ+ terminology varies across papers; we cite the language and/or abbreviation adopted in the individual papers. LGB = lesbian, gay, and bisexual. LGBT = lesbian, gay, bisexual, and transgender. LGBTQ = lesbian, gay, bisexual, transgender, and queer. LGBTQ+ = lesbian, gay, bisexual, transgender, and queer others who are a gender and sexual minority (i.e., +). RCT = randomized controlled trial. SGMYs = sexual and gender minority youths. ^b^ Where a mean age was not provided or could not be calculated, the age range reported or confirmation from the paper’s corresponding author was used to determine that the mean age was ≤19 years.

**Table 2:** Common content across the intervention-focused studies.

Technique or Coping Strategy [Category Type]	Intervention/s Where This Was Utilized	Example Description
Relaxation exercises [behavioral]	A mental health promotion program (i.e., [24]), “SPARX” and “Rainbow SPARX”	Relaxation exercises included “diaphragmatic breathing and progressive muscle relaxation” [24] (p. 11), although the exercises were not adapted in any way to better meet the needs of SGMYs.
Behavioral activity/ activation [behavioral]	“AFFIRM”, a culturally adapted intervention (i.e., [23]), “Project YES”, “SPARX”, and “Rainbow SPARX”	Key messages to SGMYs included: “…the fewer pleasant activities people do, the more depressed they feel…[address this by]…engaging in activities that are pleasant, rewarding, and inspiring” [23] (p. 897), e.g., dancing (even if SGMY’s family is unsupportive of “gay” activities) [23].
Problem solving [cognitive/emotional]	“ASSET”, an attachment-based intervention (i.e., [26]), a culturally adapted intervention (i.e., [23]), a mental health promotion program (i.e., [24]), “SPARX”, and “Rainbow SPARX”	Problem solving introduced using “…STEPS (Say what the problem is, Think of solutions, Examine these ideas, Pick one and try it, See what happens)” [42] (p. 207) using problems of relevance (e.g., worrying friends will reject an SGMY when they come out) [42].
Enhancing supports [social/environmental]	“AFFIRM”, “ASSET”, a culturally adapted intervention (i.e., [23]), and an unnamed intervention (i.e., [25])	Skills were taught and practiced, e.g., “…using education and rehearsal within an affirmative context that…enhances connection to and support from peer and adult allies…” [34] (p. 138), such as identifying a plan for building a supportive social network for SGMYs [34].
Psycho-education [cognitive/emotional]	“AFFIRM”, a mental health promotion program (i.e., [24]), and “Rainbow SPARX”	Specific examples included highlighting “…the connection between experiencing a stressor, emotional reactions, and behavioral responses” [24] (p. 11) and “Understanding the impact of anti-LGBTQ attitudes and behaviors on stress” [34] (p. 139).
Recognizing problematic cognitions [cognitive]	“AFFIRM”, “SPARX”, “Rainbow SPARX”, and an unnamed intervention (i.e., [25])	SGMY-specific examples of recognizing problematic cognitions were highlighted, for instance, “Someone gives you grief because you’re different. Here comes the [possible] negative thought: “I’m a freak and no one will ever love me”…” [42] (p. 208).
Cognitive restructuring [cognitive]	“AFFIRM”, a culturally adapted intervention (i.e., [23]), a mental health promotion program (i.e., [24]), “Rainbow SPARX”, “SPARX”, and an unnamed intervention (i.e., [25])	ABCD method used (example provided for “I am genderqueer”) “A: is the Activating event…B: is the Belief or the thought that you are having…C: is the Consequence of your thought…D: is the way in which you Dispute or talk back to your thought” [33] (p. 5).
Building family relationships [social/environmental]	An attachment-based intervention (i.e., [26]) and “Familias con Orgullo”	An example included parents using “…newly learned communication skills and practice with adolescents by discussing a relevant issue in the youth’s life related to being a sexual minority” [40] (p. 7).
Educating families [social/environmental]	An attachment-based intervention (i.e., [26]), “Familias con Orgullo”, and an unnamed intervention [30]	Importance of education reinforced, such as having “…written material that the parents could read to educate themselves about many different aspects of sexual minority life, including things as simple as definitions and as complicated as legislative issues…” [30] (p. 184).
Raising awareness of resources [cognitive]	“Singularities” and “Q-Chat Space”	Digital resources were highlighted by SGMYs: “Youth also discussed increased consumption of digital media (e.g., video, games, music), particularly identity-specific online content…They frequently exchanged content recommendations” [37] (p. 451).
Public narratives [cognitive and social/environmental]	“Brave Trails” and “Rainbow YOUTH workshops”	Developing and sharing narratives (e.g., coming out experiences) to support positive change seen as especially useful: “… [An] exercise in public narrative[s], that is, articulating a “story of self” to promote social change or advocacy goals” [39] (p. 371).
Peer support for SGMYs [social/environmental]	“AFFIRM”, “ASSET”, “Brave Trails”, “Q-Chat Space”, and “Hatch Youth”, as well as an unnamed intervention and evaluation of LGBT-related school interventions/resources (i.e., [25,29]) regarding gay–straight alliances	Peer support valuable: “…[Hatch Youth includes] a youth-led peer support group where participants talk about the events and issues in their lives and/or process a specific topic…[including] self-awareness and acceptance, coming out…” [45] (p. 360).

**Table 3:** Overview of non-intervention-focused studies (n = 44).

Study Author/s (Year) [citation]	Aim of the Study (Method/s)	Sample Size	Population (Age Range) [Mean Age] ^a^	Country or Countries	LGBTQ+ Terminology Used and Focus ^b^	Key Reported Findings
Austin et al., (2020) [59]	To explore what helps promote wellbeing and protects transgender and gender diverse youths (TGDs) against psychological distress (qualitative study)	n = 260	Adolescents and young adults (14–22 years) [17.3 years]	Canada and USA	TGD	The Internet is “life saving” (p. 37)—it is where transgender youths can heal, grow, and thrive. Online, TGD youths can escape stigma and violence, experience belonging, build confidence, feel hopeful, and there are opportunities for “giving back” to others.
Berger et al., (2021) [55]	To investigate ways LGBTQ adolescents make use of social media for exploring their identity and seek support from other LGBTQ peers (qualitative study)	n = 30	Adolescents (14–17 years) [16.2 years]	Australia	LGBTQ youths	Social media assists identity development, relationships, and supports wellbeing, but is not always free of discrimination. Facebook groups allow for a connection with LGBTQ peers, and social media was considered a vital support for those with mental health problems, including suicidal ideation.
Bond and Loewenster (2014) [79]	To quantify what makes LGB youths happy and to examine the content of their happy memory narratives and other variables associated with LGB adolescents’ wellbeing (mixed methods)	n = 390	Adolescents (13–19 years) [16.5 years]	USA	LGB	Happy memory narratives are important in terms of overall wellbeing and 77% of participants described one that was either everyday leisure or a special occasion, and 71% included some mention of friends. Few recalled LGB-specific events as happy memories (e.g., taking part in a pride parade).
Budge et al., (2018) [50]	To explore how trans youths managed exploring their gender identity, coming out to others, and navigated environments and society (qualitative study)	n = 20	Children and adolescents (7–18 years) [12.2 years]	USA	Trans youths	Six themes related to coping with gender identity were identified—negotiating gender, avoidance, emotional relief, personal solace, support, and active engagement. The same coping strategy could be either harmful or useful, depending on the timing, purpose, and context.
Budge et al., (2021) [77]	An exploration of how transgender and gender nonconforming children and adolescents (TGNCs) understand, experience, and label emotional experiences (qualitative study)	n = 20	Children and adolescents (7–18 years) [12.2 years]	USA	TGNCs	Youths struggle with what the future entails when their “mental energy is focused on coping with current stressors” (p. 162). There is a lack of adult transgender role models. Apathy appeared to be used as a possible defense against emotional pain. It is important to highlight pleasant emotions when these emotions are experienced.
Butler and Astbury (2008) [67]	An exploration of the meaning of coming out in relation to South Africa’s gay and lesbian youths in post-apartheid South Africa (qualitative study)	n = 18	Adolescents and young adults (16–21 years) [≤19 years]	South Africa	Gay and lesbian youths	Defense mechanisms identified by the researchers (e.g., denial, avoidance, compartmentalization, suppression, compensation, sublimation, undoing, rationalization, and intellectualization). A common coping strategy is “learning to hide” (p. 233), but keeping distance can lead to isolation.
Craig et al., (2015) [51]	To describe media and their influence on the resilience of LGBTQ young people (qualitative study)	n = 19	Adolescents and young adults (18–22 years) [≤19 years]	Canada	LGBTQ	Four themes were identified where media-use enabled: “…coping through escapism; feeling stronger; fighting back; and finding and fostering community…” (p. 254). For example, a participant highlighted that “…media is a form of escapism from the harsh reality that is the heteronomative, the heterosexist world that we live in…” (p. 262).
Craig et al., (2017) [61]	To explore the experiences of stress and resilience amongst ethno-racial and sexual minority girls (ESMGs) (qualitative study)	n = 40	Adolescents (15–18 years) [16.0 years]	USA	ESMGs	Resilience can be manifested asa young person serving as the family’s educator, being “out” in the open with their family, and creating “pockets of safety” (p. 628). For instance, participants “…deftly negotiated complicated and adversarial religious perspectives to create safe spiritual experiences…” (p. 628).
Craig et al., (2020) [60]	To determine how SGMYs manage negative comments online and understand the impact of these negative comments in terms of the wellbeing of SGMYs (mixed methods)	n = 5243	Adolescents and young adults (14–29 years) [18.2 years]	Canada and USA	Sexual and gender minority youths (SGMYs)	Themes—appraising the situation/themselves; avoiding (e.g., ignoring comments); responding (e.g., fighting back); adaptive coping (e.g., seeking and/or providing support); maladaptive coping (e.g., self-harming); impacting wellbeing (e.g., feeling distressed or tired); and a non-issue/do not experience this.
Craig et al., (2021) [58]	The development of a social media benefits scale (SMBS) for LGBTQ+ young people (quantitative study)	n = 6178	Adolescents and young adults (14–29 years) [18.2 years]	USA and Canada	LGBTQ+	The benefits of social media use for LGBTQ+ youths include opportunities for emotional support and development; general education; entertainment; and obtaining identity-specific information. Younger participants were more likely to use social media for beneficial factors than older youth.
Davis, Saltzburg, and Locke (2009) [80]	An exploration of the emotional and psychological needs of GLBT youths and an assessment of support systems and their current gaps (mixed methods)	n = 33	Adolescents and young adults (14–23 years) [18.5 years]	USA	GLBT	Participants identified many issues related to improving environments to enhance their psychological and physical safety. “…GLBT-focused youth centers appear to offer layers of protection for youth in various forms….” (p. 1040), primarily due to their ability to support a connection with similar peers.
Davis, Saltzburg, and Locke (2010) [81]	To use concept mapping to explore the psychosocial support needs of GLBTQ youths (quantitative study)	n = 20	Adolescents and young adults (14–23 years) [18.0 years]	USA	GLBTQ youths	Three primary areas identified—developing protective supports (because GLBTQ youths feel “unprotected, vulnerable, and invalidated” p. 244); mental health-related supports are required; and these need to be culturally relevant services. Teaching youths how to effectively self-advocate to enhance supports is also important.
Dewaele et al., (2013) [68]	An exploration of how visibility management can function as a coping strategy tied to their minority stress experiences (qualitative study)	n = 24	Adolescents (16–18 years) [≤19 years]	Belgium (Flanders)	LGB	LGB youths handle the visibility of their minority status differently, depending on the context. Being “closed” can reduce “…external stressors, such as verbal aggression and discrimination…” (p. 692), but then risks exposure to internal stressors, such as the fear of being “caught” and feeling dishonest.
DiFulvio (2011) [47]	The meaning of social connection and its importance for resilience when working with sexual minority youths (SMYs) (qualitative study)	n = 15	Adolescents and young adults (14–22 years) [18.0 years]	USA	SMYs	Connectedness is key, themes identified: affirming the self; finding others similar to you; and moving toward action. Connection recognized for its potential allowing “…one to reach beyond the self, take action against his/her own oppression and situates…[this] into a larger collective struggle” (p. 1616).
Erhard and Ben-Ami (2016) [57]	To determine what could assist LGB secondary school students to cope with school-based homophobic bullying (qualitative study)	n = 20	Adolescents (15–18 years) [17.0 years]	Israel	LGB	Five main coping mechanisms to manage school homophobic bullying identified: cognitive appraisals of their school’s anti-LGB incidents; assertive communication; becoming an LGB community advocate; tactical ignoring; and questioning and resisting rigid (and culturally bound) sexuality labels.
Follins (2011) [65]	An exploration of how young Black lesbians manage their multiple oppressed identities (qualitative study)	n = 10	Adolescents and young adults (16–20 years) [18.0 years]	USA	Young Black lesbians	Black LGB peers important, as participants derived a “…sense of comfort with other black LGB people; they could escape homophobia; and it decreased their social isolation….” (p. 376). Difficult when the participant did not know or had few Black lesbian peers. There is a need to address multiple-identity management.
Gibbs and Goldbach (2021) [66]	An exploration of the negative messages that sexual minority adolescents (SMAs) receive from religious sources, and the strategies used to make sense of these messages (qualitative study)	n = 46	Adolescents (14–19 years) [16.3 years]	USA	SMAs	Anti-homosexual religious messages focus on “creation, sin, and afterlife” (p. 2189). Coping strategies (cognitive)—using religious identity material or sexual minority identity content to reduce the negative impacts, adding new information to invalidate the messages, and distancing oneself from or rejecting the actual message.
Goldbach and Gibbs (2015) [52]	The study aimed to identify the coping strategies, responses, and resources of sexual minority adolescents (SMAs) in terms of stress management (qualitative study)	n = 48	Adolescents (14–19 years) [16.3 years]	USA	SMAs	Coping strategies—“Voluntary Engagement” (e.g., time within LGBTQ+ community), “Voluntary Disengagement” (e.g., not coming out to others), “Involuntary Engagement” (e.g., using religious beliefs to build confidence), “Involuntary disengagement” (e.g., apathy), and “Coping Resources”.
Goldbach and Gibbs (2017) [53]	An exploration of whether the minority stress theory applies to sexual minority adolescents (SMAs) (qualitative study)	n = 48	Adolescents (14–19 years) [16.3 years]	USA	SMAs	Coping varied from “…LGBT connections (e.g., going to LGBT pride events, using LGBT online resources, going to an LGBT youth center, becoming involved in a gay–straight alliance) to conforming to heteronormative behaviors (e.g., dating individuals of the opposite sex….” p. 42).
Grossman, D’augelli, and Frank (2011) [82]	An exploration of mental health problems and their relationship to aspects of psychological resiliency (quantitative study)	n = 55	Adolescents and young adults (15–21 years) [≤19 years]	USA	Transgender youths	The more gender non-conforming a young person is, the more abuse they receive. Higher self-esteem, a higher sense of personal mastery, and greater perceived social support predicted positive mental health outcomes for transgender youths.
Higa et al., (2014) [83]	To determine the factors associated with LGBTQ youths’ wellbeing from the youths’ perspectives (qualitative study)	n = 68	Adolescents and young adults (14–24 years) [≤19 years]	USA	LGBTQ	Positive factors that enhanced wellbeing were linked to supporting LGBTQ youths’ own identity development, peer networks, and involvement in the LGBTQ community (although this is lacking in rural areas—so online supports are especially valuable).
Jessen et al., (2021) [84]	To explore the subjective experiences of gender dysphoria among help-seeking transgender and gender nonconforming (TGNC) youths (qualitative study)	n = 15	Adolescents (13–19 years) [16.0 years]	Norway	TGNC youths	Participants strived to “…reach a state of feeling whole, where they can ‘just be themselves’” (p. 3498). Their commitment to a male identity transformed their relationship with their bodies and “…made the participants feel whole and complete” (p. 3498), but this could lead to new forms of gender dysphoria.
Johns et al., (2021) [46]	To examine the in-school experiences of transgender youths and understand their coping strategies, in order to identify opportunities for improving schools (qualitative study)	n = 8	Adolescents (15–19 years) [17.3 years]	USA	Transgender youths	Coping included—transgender youth fostering inclusion (e.g., “What I used to do, I would go up before class and make sure they knew to call by my right name if it was a sub [substitute teacher]” p. 887) and taking steps toward fostering social connections (i.e., intentional actions to connect to alleviate the impacts of stressors).
Johnson et al., (2020) [73]	To better understand the conditions under which trans adolescents perceive specific parental behaviors as being supportive of or rejecting them (qualitative study)	n = 24	Adolescents and young adults (16–20 years) [17.8 years]	USA	Trans adolescents	To cope with parental rejection, some participants described engaging in self-harm behaviors. When trans adolescents have parents exhibiting rejecting behaviors, family work will be important, and if not viable then “…attempts should be made to connect youth to other forms of social support…” (p. 167).
Kuper, Coleman and Mustanski (2014) [78]	To examine how racial–ethnic minority LGBT youths cope with both racial–ethnic as well as LGBT-related stresses (mixed methods)	n = 213	Adolescents and young adults (16–20 years) [18.3 years]	USA	LGBT youths of color	Multiple cognitive and behavioral strategies identified, e.g., “preparation for future bias or harassment”, being “cautious, guarded, or less trusting”, attempt to “ignore or not be affected by other’s views or reactions”, “be or focus on oneself”, and “take care of self and problems” (p. 712).
Madsen and Green (2012) [71]	To better understand the specific ways gay adolescent males successfully cope with prejudice, discrimination, and stigma (qualitative study)	n = 8	Adolescents (15–18 years) [16.6 years]	USA	Gay-identified male adolescents	Coping themes related to thoughts and feelings (e.g., “Regulation of immediate emotional reaction in context of situation” p. 146 and “Analysis of the anti-LGB incident for personal relevance and severity” p. 147) and actions or behaviors (e.g., engaging in distractions, such as sports and music).
Marshall et al., (2015) [85]	To examine the bullying experiences of sexual minority youths (SMYs) in a rural area (qualitative study)	n = 16	Adolescents and young adults (15–20 years) [18.0 years]	USA	SMYs	Peers, family members, personnel at school, and youth services (or a combination of these) formed critical types of support. SMYs found supportive staff at school to cope with bullying and it was suggested that “…if you don’t have a support network, if you don’t have anybody [you should] find somebody” (p. 338).
McDermott, Hughes, and Rawlings (2018) [75]	To examine the circumstances in which LGBTQ+ young people seek help for suicidal feelings and self-harming (mixed methods)	n = 818	Adolescents and young adults (13–25 years) [18.6 years]	UK	LGBTQ	Participants only asked for help when at a crisis point. Reluctance to seek help related to “…negotiating sexuality, gender, mental health and age norms; being unable to talk about emotions; and coping and self-reliance” (p. 156). Some perceived self-harming as a positive coping strategy for managing stress.
McDermott, Roen, and Scourfield (2008) [62]	To explore the connections between sexual identities and self-destructive behaviors in LGBT young people (qualitative study)	n = 27	Adolescents and young adults (16–25 years) [≤19 years]	UK	LGBT	Common strategies to manage mistreatment included “…routinization and minimizing of homophobia; maintaining individual ‘adult’ responsibility; and constructing “proud” identities….” (p. 820). Self-harming, in particular, cutting, could be perceived as a coping strategy for when individuals are very distressed.
McInroy (2020) [64]	To investigate online fandom communities as supports for SGMYs, and their potential to contribute to the resilience and positive adjustment of SGMYs (mixed methods)	n = 3665	Adolescents and young adults (14–29 years) [17.8 years]	USA and Canada	SGMYs	Fandoms/online fan groups can assist SGMYs by increasing connectedness, providing opportunities for support or mentorship, facilitating the navigation of challenges, and encouraging feelings of strength. For instance, these groups help SGMYs to “cope with real life…[and] feel better” about themselves or their situation (p. 1882).
O’Brien, Parra, and Cederbaum (2021) [72]	An exploration of the self-care practices of sexual minority adolescents (SMAs) during the COVID-19 pandemic (qualitative study)	n = 770	Adolescents (15–19 years) [17.5 years]	USA	SMAs	Key strategies used during COVID-19—“relationships” (e.g., spending time with others online), setting “routines”, “body and mind” (e.g., exercise and meditation), “rest and reset” (e.g., art and reading), and “tuning out” (e.g., binge-watching TV) (p. 1053). Alcohol and drugs also cited as a strategy.
Rubin and McClelland (2015) [69]	To explore the phenomenon of sexual identity management and the psychological costs of monitoring Facebook content (qualitative study)	n = 8	Adolescents (16–19 years) [17.4 years]	USA	Queer young women of color	Participants developed relationships and support via Facebook, which requires sharing (e.g., thoughts, behaviors, and ideas), but, at times, they needed to hide and silence their emerging sexuality. The tempering of self-presentation, to offset possible social exclusion, was ongoing and perceived as treacherous.
Scourfield, Roen, and McDermott (2008) [74]	To examine how LGBT young people think about suicide and self-harm as well as identify the strategies they employ when distressed (qualitative study)	n = 69	Adolescents and young adults (16–25 years) [≤19 years]	UK	LGBT	Coping strategies were categorized as resilient (e.g., drawing strength from resisting discrimination), managing ambivalence (e.g., being “…”out and proud”, but also simultaneously uncomfortable with their sexuality or despising aspects of gay culture” p. 332), and engaging in self-destructive behavior (e.g., cutting).
Selkie et al., (2020) [56]	To explore how transgender adolescents use social media for social support (qualitative study)	n = 25	Adolescents (15–18 years) [16.0 years]	USA	Transgender adolescents	Online strategies identified included support received from transgender-related online communities (including emotional support via peers and role models), “appraisal support for validating their experiences” (p. 275), and informational support for health decision-making and for educating others.
Singh (2013) [86]	Examined the experiences of resilience transgender youths of color described as they negotiated the intersections of transprejudice as well as racism (qualitative study)	n = 13	Adolescents and young adults (15–24 years) [19.0 years]	USA	Transgender youths of color	Daily lived experience of resilience despite racism and transprejudice encapsulated—“evolving, simultaneous self-definition of racial/ethnic and gender identities” (p. 690), an awareness of adultism (i.e., dominance of youth by adults), self-advocacy, finding a place within the LGBTQ community, and using social media to affirm this.
Steinke et al., (2017) [87]	To assess the issues most important to sexual and gender minority youths (SGMYs) that are least likely to be met by existing resources (qualitative study)	n = 92	Adolescents and young adults (15–20 years) [17.0 years]	USA	SGMYs	SGMYs search for supportive, validating communities and relevant, accurate information online. Online resources should represent diverse identities, be comprehensive and link to both mental and sexual health, and not be crisis oriented (i.e., not be solely risk focused, but instead address health holistically).
Strauss et al., (2019) [70]	To explore the perspectives of trans and gender diverse (TGD) young people in relation to utilizing digital technologies to improve their mental health (qualitative study)	n = 14	Adolescents (11–18 years) [15.6 years]	Australia	TGD young people	Online forms of support include diversionary activities (e.g., games). Apps and digital resources are valuable when they include social elements and/or teach skills (e.g., mental health management and self-care). Chat/email services useful because of their availability outside of office hours and ability to maintain privacy.
Toomey and Anhalt (2016) [76]	Examined mindfulness as a coping strategy for bias-based school victimization (quantitative study)	n = 236	Adolescents and young adults (14–24 years) [19.0 years]	USA	Latina/o sexual minority students	Mindful responses, e.g., “I am aware that I am upset because I am encountering discrimination” (p. 434) versus shameful or judgmental responses, e.g., “I’m being discriminated against. Something must be wrong with me” (p. 434). High levels of mindfulness protective for sexuality-based, but not ethnicity-based, victimization.
Torres et al., (2012) [63]	Examined natural mentoring relationships amongst gay, bisexual, and questioning (GBQ) males (qualitative study)	n = 39	Adolescents and young adults (15–22 years) [19.0 years]	USA	GBQ males	Most participants could identify a natural mentor (e.g., teacher, school nurse, or neighbor). More experienced and knowledgeable GBQ peers seen as valuable for support. Social supports to ideally encompass emotional, instructional, and informational elements, as well as unconditional acceptance.
Wagaman et al., (2019) [48]	An exploration of how transgender and gender-expansive (TGE) youths and young adults make sense of both their challenges and successes (qualitative study)	n = 85	Adolescents and young adults (13–24 years) [18.0 years]	USA	TGE youths and young adults	“Buffers to Destabilization” (p. 56) important, e.g., connection to others similar to them (and the wider LGBTQ+ community). Other strategies included intentionally disconnecting from environments and people that were not good for them, and personal growth—“…an inwardly focused process and capacity…described as strengthening…” (p. 7).
Wike et al., (2021) [54]	Explored the victimization experiences of rural LGBTQ+ youths, their supports, and the ways they demonstrate resilience (qualitative study)	n = 11	Adolescents and young adults (12–21 years) [16.0 years]	USA	LGBTQ+	Social media enabled connectivity and created a sense of community for rural LGBTQ+ youths (and a way to come out to many people at a distance). They could receive affirming messages that fostered belonging. Collective resilience important, e.g., “…the gay youth of [this town]… just amazing. We’re strong and we’re powerful” (p. 11).
Winskell and Sabben (2016) [22]	To identify the contextual factors that inform sexual stigma and the cultural meanings that underpin this stigma (qualitative study)	n = 56	Adolescents and young adults (13–24 years) [19.0 years]	10 African countries	Same-sex attraction (but % same-sex attracted not determined)	Alcohol and drug use a possible coping strategy. Examples of strategies to manage included the use of secrecy and concealment. Increased visibility was a potential problem for same-sex-attracted young Africans. The use of stories (and other narratives) enabled access to a diverse range of youths.
Wolowic et al., (2017) [88]	An exploration of how LGBTQ youths recognize and deploy symbols of support (qualitative study)	n = 66	Adolescents (14–19 years) [16.6 years]	USA and Canada	LGBTQ	LGBT youths displayed rainbow symbols to disclose their community affiliation to others (e.g., to family members and authority figures). This symbol was associated with positive emotions, memories, and aspirations. There were learned meanings associated with rainbow symbols and these assisted them to navigate toward supports.
Zeeman et al., (2017) [49]	To explore the views of transgender young people in order to determine what is needed to promote their emotional wellbeing and resilience (qualitative study)	n = 5	Adolescents (14–19 years) [≤19 years]	UK	Transgender young people	Strategies to enhance resilience involved transgender young people being “…deliberately proactive in accessing supportive educational systems” (p. 392); connecting with a trans-affirming community where they can reframe their mental health challenges; and skillfully navigating relationships with family and friends.

^a^ Where a mean age was not provided or could not be calculated, the age range reported or confirmation from the paper’s corresponding author was used to determine that the mean age was ≤19 years. ^b^ LGBTQ+ terminology varies across papers; we cite the language and/or abbreviation adopted in the individual papers. ESMG = ethno-racial and sexual minority girls. GBQ = gay, bisexual, and questioning. GLBT = gay, lesbian, bisexual, and transgender. GLBTQ = gay, lesbian, bisexual, transgender, and queer. LGB = lesbian, gay, and bisexual. LGBTQ = lesbian, gay, bisexual, transgender, and queer. SMA = sexual minority adolescents. SMYs = sexual minority youths. TGDs = transgender and gender diverse youths. TGE = transgender and gender-expansive. TGNC = transgender and gender non-conforming.

## Data Availability

Not applicable.

## Appendix A. Databases, Search Terms, and Search Results

### Appendix A.1. Medline

Search period: From inception in 1946 to 19 January 2022

Electronic limits: “Humans”, “Adolescent (13 to 18 years)”, and the “English” language

Results: 1100 articles

**Table A1:** Medline search terms.

Sexual and Gender Minority		Psycho-Social Coping Strategies
“Sexual and Gender Minorities”/OR gender minorit*.mp. OR LGB*.mp. OR sexual minorit*.mp. OR gender identit*.mp. OR gender varianc*.mp. OR gender?queer.mp. OR queer*.mp. OR lesbian*.mp. OR gay*.mp. OR bisexual*.mp. OR transgender*.mp. OR transsexual*.mp. OR homosexual*.mp. OR non?binary.mp.	AND	Coping*.mp. OR Cope.mp. OR Adaptive.mp. OR Self-Management/ OR Self Care/ OR Mental Health/ OR “Resilience, Psychological”/OR mental well?being.mp. OR self help.mp. OR “Psychotherapy”/

Note: * The truncation symbol; ? The wildcard symbol; “ ”/ A subject heading within the database; .mp. The searched term can be identified from multiple places, for example, the paper’s title, abstract, subject headings, or keywords.

### Appendix A.2. Embase

Search period: From inception in 1980 to 19 January 2022

Electronic limits: “Humans”, “Adolescent (13 to 17 years)”, and the “English” language

Results: 1226 articles

**Table A2:** Embase search terms.

Sexual and Gender Minority		Psycho-Social Coping Strategies
gender minorit*.mp. OR LGB*.mp. OR sexual minorit*.mp. OR gender identit*.mp. OR gender varianc*.mp. OR gender?queer.mp. OR “LGBTQIA+ people”/OR queer*.mp. OR “Sexual and Gender Minority”/OR lesbian*.mp. OR “Homosexual Female”/OR gay*.mp. OR “Bisexuality”/OR bisexual*.mp. OR transgender*.mp. OR “Transgender”/OR transsexual*.mp. OR homosexual*.mp. OR “Homosexuality”/OR non?binary.mp.	AND	coping*.mp. OR cope.mp. OR adapat*.mp. OR “Self Care”/OR self management.mp. OR “Mental Health”/OR resilience.mp. OR “Psychological Well-Being”/OR mental well?being.mp. OR “Self Help”/OR “Psychotherapy”/

Note: * The truncation symbol; ? The wildcard symbol; “ ”/ A subject heading within the database; .mp. The searched term can be identified from multiple places, for example, the paper’s title, abstract, subject headings, or keywords.

### Appendix A.3. PsycInfo

Search period: From inception in 1806 to 19 January 2022

Electronic limits: “Humans”, “Adolescent (13 to 17 years)”, and the “English” language

Results: 1366 articles

**Table A3:** PsycInfo search terms.

Sexual and Gender Minority		Psycho-Social Coping Strategies
sexual and gender minorities.mp. OR gender minorit*.mp. OR LGB*.mp. OR sexual minorit*.mp. OR gender minorit*.mp. OR gender varianc*.mp. OR gender?queer.mp. OR queer*.mp. OR lesbian*.mp. OR gay*.mp. OR bisexual*.mp. OR transgender*.mp. OR transsexual*.mp. OR homosexual*.mp. OR non?binary.mp.	AND	Coping*.mp. OR Cope.mp. OR Adaptive.mp. OR self-management.mp. OR self care.mp. OR mental health.mp. OR “Resilience (Psychological)”/OR “Psychotherapy”/OR “Well Being”/OR “Self-Help Techniques”/

Note: * The truncation symbol; ? The wildcard symbol; “ ”/ A subject heading within the database; .mp. The searched term can be identified from multiple places, for example, the paper’s title, abstract, subject headings, or keywords.

## References

[1] Lucassen M.F.G., Stasiak K., Samra R., Frampton C.M., Merry S.N. (2017). Sexual minority youth and depressive symptoms or depressive disorder: A systematic review and meta-analysis of population-based studies. Aust. N. Z. J. Psychiatry.

[2] Reisner S.L., Poteat T., Keatley J., Cabral M., Mothopeng T., Dunham E., Holland C.E., Ryan M., Baral S.D. (2016). Global health burden and needs of transgender populations: A review. Lancet.

[3] Clark T.C., Lucassen M.F.G., Bullen P., Denny S.J., Fleming T.M., Robinson E.M., Rossen F.V. (2014). The health and well-being of transgender high school students: Results from the New Zealand Adolescent Health Survey (Youth’12). J. Adolesc. Health.

[4] Meyer I.H. (2003). Prejudice, social stress, and mental health in lesbian, gay, and bisexual populations: Conceptual issues and research evidence. Psychol. Bull..

[5] Denny S., Lucassen M.F.G., Stuart J., Fleming T., Bullen P., Peiris-John R., Rossen F.V., Utter J. (2016). The association between supportive high school environments and depressive symptoms and suicidality among sexual minority students. J. Clin. Child Adolesc. Psychol..

[6] Lucassen M.F.G., Samra R., Rimes K.A., Brown K.E., Wallace L.M. (2022). Promoting resilience and well-being through co-design (The PRIDE Project): Protocol for the development and preliminary evaluation of a prototype resilience-based intervention for sexual and gender minority youth. JMIR Res. Protoc..

[7] Government Equalities Office (2018). LGBT Action Plan: Improving the Lives of Lesbian, Gay, Bisexual and Transgender People.

[8] Gilbey D., Morgan H., Lin A., Perry Y. (2020). Effectiveness, acceptability, and feasibility of digital health interventions for LGBTIQ+ young people: Systematic review. J. Med. Internet Res..

[9] Hobaica S., Alman A., Jackowich S., Kwon P. (2018). Empirically based psychological interventions with sexual minority youth: A systematic review. Psychol. Sex. Orientat. Gend. Divers..

[10] Van Der Pol-Harney E., McAloon J. (2019). Psychosocial interventions for mental illness among LGBTQIA youth: A PRISMA-based systematic review. Adolesc. Res. Rev..

[11] National Institute for Health and Care Excellence (2019). Depression in Children and Young People: Identification and Management [NICE Guideline NG134].

[12] Foy A.A., Morris D., Fernandes V., Rimes K.A. (2019). LGBQ+ adults’ experiences of Improving Access to Psychological Therapies and primary care counselling services: Informing clinical practice and service delivery. Cogn. Behav. Ther..

[13] Rimes K.A., Ion D., Wingrove J., Carter B. (2019). Sexual orientation differences in psychological treatment outcomes for depression and anxiety: National cohort study. J. Consult. Clin. Psychol..

[14] Lucassen M.F.G., Stasiak K., Fleming T., Frampton C., Perry Y., Shepherd M., Merry S.N. (2021). Computerized cognitive behavioural therapy for gender minority adolescents: Analysis of the real-world implementation of SPARX in New Zealand. Aust. N. Z. J. Psychiatry.

[15] Lucassen M.F.G., Samra R., Iacovides I., Fleming T., Shepherd M., Stasiak K., Wallace L. (2018). How LGBT+ young people use the internet in relation to their mental health and envisage the use of e-therapy: Exploratory study. JMIR Serious Games.

[16] Christmas C.M., Khanlou N. (2019). Defining youth resilience: A scoping review. Int. J. Ment. Health Addict..

[17] Colpitts E., Gahagan J. (2016). The utility of resilience as a conceptual framework for understanding and measuring LGBTQ health. Int. J. Equity Health.

[18] McDermott E., Nelson R., Weeks H. (2021). The politics of LGBT+ health inequality: Conclusions from a UK scoping review. Int. J. Environ. Res. Public Health.

[19] Gahagan J., Colpitts E. (2017). Understanding and measuring LGBTQ pathways to health: A scoping review of strengths-based health promotion approaches in LGBTQ health research. J. Homosex..

[20] Tricco A.C., Lillie E., Zarin W., O’Brien K.K., Colquhoun H., Levac D., Moher D., Peters M.D.J., Horsley T., Weeks L. (2018). PRISMA extension for scoping reviews (PRISMA-ScR): Checklist and explanation. Ann. Intern. Med..

[21] Arksey H., O’Malley L. (2005). Scoping studies: Towards a methodological framework. Int. J. Soc. Res. Methodol..

[22] Winskell K., Sabben G. (2016). Sexual stigma and symbolic violence experienced, enacted, and counteracted in young Africans’ writing about same-sex attraction. Soc. Sci. Med..

[23] Duarté-Vélez Y., Bernal G., Bonilla K. (2010). Culturally adapted cognitive-behavioral therapy: Integrating sexual, spiritual, and family identities in an evidence-based treatment of a depressed Latino adolescent. J. Clin. Psychol..

[24] Heck N.C. (2015). The potential to promote resilience: Piloting a minority stress-informed, GSA-based, mental health promotion program for LGBTQ youth. Psychol. Sex. Orientat. Gend. Divers..

[25] Craig S.L., Austin A., Alessi E. (2013). Gay affirmative cognitive behavioral therapy for sexual minority youth: Clinical adaptations and approaches. Clin. Soc. Work. J..

[26] Diamond G.M., Diamond G.S., Levy S., Closs C., Ladipo T., Siqueland L. (2013). Attachment-based family therapy for suicidal lesbian, gay, and bisexual adolescents: A treatment development study and open trial with preliminary findings. Psychol. Sex. Orientat. Gend. Divers..

[27] Craig S.L., Austin A., McInroy L.B. (2014). School-based groups to support multiethnic sexual minority youth resiliency: Preliminary effectiveness. Child Adolesc. Soc. Work. J..

[28] Lucassen M.F.G., Burford J. (2015). Educating for diversity: An evaluation of a sexuality diversity workshop to address secondary school bullying. Australas. Psychiatry.

[29] Greytak E.A., Kosciw J.G., Boesen M.J. (2013). Putting the “T” in “resource”: The benefits of LGBT-related school resources for transgender youth. J. LGBT Youth.

[30] Grafsky E.L., Gary E.A. (2018). What sexual minority youths want in a program to assist with disclosure to their family. J. Gay Lesbian Soc. Serv..

[31] Egan J.E., Corey S.L., Henderson E.R., Abebe K.Z., Louth-Marquez W., Espelage D., Hunter S.C., DeLucas M., Miller E., Morrill B.A. (2021). Feasibility of a web-accessible game-based intervention aimed at improving help seeking and coping among sexual and gender minority youth: Results from a randomized controlled trial. J. Adolesc. Health.

[32] Austin A., Craig S.L. (2015). Empirically supported interventions for sexual and gender minority youth. J. Evid.-Inf. Soc. Work..

[33] Austin A., Craig S.L., D’Souza S.A. (2018). An AFFIRMative cognitive behavioral intervention for transgender youth: Preliminary effectiveness. Prof. Psychol. Res. Pract..

[34] Craig S.L., Austin A. (2016). The AFFIRM open pilot feasibility study: A brief affirmative cognitive behavioral coping skills group intervention for sexual and gender minority youth. Child. Youth Serv. Rev..

[35] Craig S.L., Austin A., Huang Y.T. (2018). Being humorous and seeking diversion: Promoting healthy coping skills among LGBTQ+ youth. J. Gay Lesbian Ment. Health.

[36] Coulter R.W.S., Sang J.M., Louth-Marquez W., Henderson E.R., Espelage D., Hunter S.C., DeLucas M., Abebe K.Z., Miller E., Morrill B.A. (2019). Pilot testing the feasibility of a game intervention aimed at improving help seeking and coping among sexual and gender minority youth: Protocol for a randomized controlled trial. JMIR Res. Protoc..

[37] Fish J.N., McInroy L.B., Paceley M.S., Williams N.D., Henderson S., Levine D.S., Edsall R.N. (2020). “I’m kinda stuck at home with unsupportive parents right now”: LGBTQ youths’ experiences with COVID-19 and the importance of online support. J. Adolesc. Health.

[38] Fish J.N., Williams N.D., McInroy L.B., Paceley M.S., Edsall R.N., Devadas J., Henderson S.B., Levine D.S. (2021). Q Chat Space: Assessing the Feasibility and Acceptability of an Internet-Based Support Program for LGBTQ Youth. Prev. Sci..

[39] Gillig T.K., Miller L.C., Cox C.M. (2019). “She finally smiles... for real”: Reducing depressive symptoms and bolstering resilience through a camp intervention for LGBTQ youth. J. Homosex..

[40] Lozano A., Estrada Y., Tapia M.I., Dave D.J., Marquez N., Baudin S., Prado G. (2021). Development of a family-based preventive intervention for Latinx sexual minority youth and their parents. Cult. Divers. Ethn. Minor. Psychol..

[41] Lucassen M.F.G., Hatcher S., Fleming T.M., Stasiak K., Shepherd M.J., Merry S.N. (2015). A qualitative study of sexual minority young people’s experiences of computerised therapy for depression. Australas. Psychiatry.

[42] Lucassen M.F.G., Merry S.N., Hatcher S., Frampton C.M.A. (2015). Rainbow SPARX: A novel approach to addressing depression in sexual minority youth. Cogn. Behav. Pract..

[43] McDanal R., Rubin A., Fox K.R., Schleider J.L. (2022). Associations of LGBTQ+ identities With acceptability and efficacy of online single-session youth mental health interventions. Behav. Ther..

[44] Painter K.R., Scannapieco M., Blau G., Andre A., Kohn K. (2018). Improving the mental health outcomes of LGBTQ youth and young adults: A longitudinal study. J. Soc. Serv. Res..

[45] Wilkerson J.M., Schick V.R., Romijnders K.A., Bauldry J., Butame S.A. (2017). Social support, depression, self-esteem, and coping among LGBTQ adolescents participating in hatch youth. Health Promot. Pract..

[46] Johns M.M., Zamantakis A., Andrzejewski J., Boyce L., Rasberry C.N., Jayne P.E. (2021). Minority stress, coping, and transgender youth in schools—Results from the Resilience and Transgender Youth Study. J. Sch. Health.

[47] DiFulvio G.T. (2011). Sexual minority youth, social connection and resilience: From personal struggle to collective identity. Soc. Sci. Med..

[48] Wagaman M.A., Shelton J., Carter R., Stewart K., Cavaliere S.J. (2019). “I’m totally transariffic”: Exploring how transgender and gender-expansive youth and young adults make sense of their challenges and successes. Child Youth Serv..

[49] Zeeman L., Aranda K., Sherriff N., Cocking C. (2017). Promoting resilience and emotional well-being of transgender young people: Research at the intersections of gender and sexuality. J. Youth Stud..

[50] Budge S.L., Belcourt S., Conniff J., Parks R., Pantalone D., Katz-Wise S.L. (2018). A grounded theory study of the development of trans youths’ awareness of coping with gender identity. J. Child Fam. Stud..

[51] Craig S.L., McInroy L., McCready L.T., Alaggia R. (2015). Media: A catalyst for resilience in lesbian, gay, bisexual, transgender, and queer youth. J. LGBT Youth.

[52] Goldbach J.T., Gibbs J. (2015). Strategies employed by sexual minority adolescents to cope with minority stress. Psychol. Sex. Orientat. Gend. Divers..

[53] Goldbach J.T., Gibbs J.J. (2017). A developmentally informed adaptation of minority stress for sexual minority adolescents. J. Adolesc..

[54] Wike T.L., Bouchard L.M., Kemmerer A., Yabar M.P. (2021). Victimization and resilience: Experiences of rural LGBTQ+ youth across multiple contexts. J. Interpers. Violence.

[55] Berger M.N., Taba M., Marino J.L., Lim M.S.C., Cooper S.C., Lewis L., Albury K., Chung K.S.K., Bateson D., Rachel Skinner S. (2021). Social media’s role in support networks among LGBTQ adolescents: A qualitative study. Sex. Health.

[56] Selkie E., Adkins V., Masters E., Bajpai A., Shumer D. (2020). Transgender adolescents’ uses of social media for social support. J. Adolesc. Health.

[57] Erhard R.L., Ben-Ami E. (2016). The schooling experience of lesbian, gay, and bisexual youth in lsrael: Falling below and rising above as a matter of social ecology. J. Homosex..

[58] Craig S.L., Eaton A.D., McInroy L.B., Leung V.W.Y., Krishnan S. (2021). Can social media participation enhance LGBTQ+ youth well-being? Development of the social media benefits scale. Soc. Media Soc..

[59] Austin A., Craig S.L., Navega N., McInroy L.B. (2020). It’s my safe space: The life-saving role of the internet in the lives of transgender and gender diverse youth. Int. J. Transgender Health.

[60] Craig S.L., Eaton A.D., McInroy L.B., D’Souza S.A., Krishnan S., Wells G.A., Twum-Siaw L., Leung V.W.Y. (2020). Navigating negativity: A grounded theory and integrative mixed methods investigation of how sexual and gender minority youth cope with negative comments online. Psychol. Sex..

[61] Craig S.L., Austin A., Alessi E.J., McInroy L., Keane G. (2017). Minority stress and HERoic coping among ethnoracial sexual minority girls: Intersections of resilience. J. Adolesc. Res..

[62] McDermott E., Roen K., Scourfield J. (2008). Avoiding shame: Young LGBT people, homophobia and self-destructive behaviours. Cult. Health Sex..

[63] Torres R.S., Harper G.W., Sanchez B., Fernandez M.I. (2012). Examining natural mentoring relationships (NMRs) among self-identified gay, bisexual, and questioning (GBQ) male youth. Child. Youth Serv. Rev..

[64] McInroy L.B. (2020). Building connections and slaying basilisks: Fostering support, resilience, and positive adjustment for sexual and gender minority youth in online fandom communities. Inf. Commun. Soc..

[65] Follins L.D. (2011). Identity development of young black lesbians in New York City: An exploratory study. J. Gay Lesbian Ment. Health.

[66] Gibbs J.J., Goldbach J.T. (2021). Religious identity dissonance: Understanding how sexual minority adolescents manage antihomosexual religious messages. J. Homosex..

[67] Butler A.H., Astbury G. (2008). The use of defence mechanisms as precursors to coming out in post-apartheid South Africa: A gay and lesbian youth perspective. J. Homosex..

[68] Dewaele A., Van Houtte M., Cox N., Vincke J. (2013). From coming out to visibility management-A new perspective on coping with minority stressors in LGB youth in Flanders. J. Homosex..

[69] Rubin J.D., McClelland S.I. (2015). ‘Even though it’s a small checkbox, it’s a big deal’: Stresses and strains of managing sexual identity(s) on Facebook. Cult. Health Sex..

[70] Strauss P., Morgan H., Toussaint D.W., Lin A., Winter S., Perry Y. (2019). Trans and gender diverse young people’s attitudes towards game-based digital mental health interventions: A qualitative investigation. Internet Interv..

[71] Madsen P.W.B., Green R.-J. (2012). Gay adolescent males’ effective coping with discrimination: A qualitative study. J. LGBT Issues Couns..

[72] O’Brien R.P., Parra L.A., Cederbaum J.A. (2021). “Trying my best”: Sexual minority adolescents’ self-care during the COVID-19 pandemic. J. Adolesc. Health.

[73] Johnson K.C., LeBlanc A.J., Sterzing P.R., Deardorff J., Antin T., Bockting W.O. (2020). Trans adolescents’ perceptions and experiences of their parents’ supportive and rejecting behaviors. J. Couns. Psychol..

[74] Scourfield J., Roen K., McDermott L. (2008). Lesbian, gay, bisexual and transgender young people’s experiences of distress: Resilience, ambivalence and self-destructive behaviour. Health Soc. Care Community.

[75] McDermott E., Hughes E., Rawlings V. (2018). Norms and normalisation: Understanding lesbian, gay, bisexual, transgender and queer youth, suicidality and help-seeking. Cult. Health Sex..

[76] Toomey R.B., Anhalt K. (2016). Mindfulness as a coping strategy for bias-based school victimization among Latina/o sexual minority youth. Psychol. Sex. Orientat. Gend. Divers..

[77] Budge S.L., Orzechowski M., Schams S., Lavender A., Onsgard K., Leibowitz S., Katz-Wise S.L. (2021). Transgender and gender nonconforming youths’ emotions: The appraisal, valence, arousal model. Couns. Psychol..

[78] Kuper L.E., Coleman B.R., Mustanski B.S. (2014). Coping with LGBT and racial-ethnic-related stressors: A mixed-methods study of LGBT youth of color. J. Res. Adolesc..

[79] Bond B.J., Loewenstern J.N. (2014). Employing memory narratives to dissect the well-being of lesbian, gay, and bisexual adolescents. J. LGBT Youth.

[80] Davis T.S., Saltzburg S., Locke C.R. (2009). Supporting the emotional and psychological well being of sexual minority youth: Youth ideas for action. Child. Youth Serv. Rev..

[81] Davis T.S., Saltzburg S., Locke C.R. (2010). Assessing community needs of sexual minority youths: Modeling concept mapping for service planning. J. Gay Lesbian Soc. Serv..

[82] Grossman A.H., D’augelli A.R., Frank J.A. (2011). Aspects of psychological resilience among transgender youth. J. LGBT Youth.

[83] Higa D., Hoppe M.J., Lindhorst T., Mincer S., Beadnell B., Morrison D.M., Wells E.A., Todd A., Mountz S. (2014). Negative and positive factors associated with the well-being of lesbian, gay, bisexual, transgender, queer, and questioning (LGBTQ) youth. Youth Soc..

[84] Jessen R.S., Waehre A., David L., Stanicke E. (2021). Negotiating gender in everyday life: Toward a conceptual model of gender dysphoria in adolescents. Arch. Sex. Behav..

[85] Marshall A., Yarber W.L., Sherwood-Laughlin C.M., Gray M.L., Estell D.B. (2015). Coping and survival skills: The role school personnel play regarding support for bullied sexual minority-oriented youth. J. Sch. Health.

[86] Singh A.A. (2013). Transgender youth of color and resilience: Negotiating oppression and finding support. Sex Roles.

[87] Steinke J., Root-Bowman M., Estabrook S., Levine D.S., Kantor L.M. (2017). Meeting the needs of sexual and gender minority youth: Formative research on potential digital health interventions. J. Adolesc. Health.

[88] Wolowic J.M., Heston L.V., Saewyc E.M., Porta C., Eisenberg M.E. (2017). Chasing the rainbow: Lesbian, gay, bisexual, transgender and queer youth and pride semiotics. Cult. Health Sex..

[89] McDermott E., Hughes E., Rawlings V. (2016). Queer Futures: Understanding Lesbian, Gay, Bisexual and Trans (LGBT) Adolescents’ Suicide, Self-Harm and Help-Seeking Behaviour (Main Results).

[90] Martell C.R., Safren S.A., Prince S.E. (2004). Cognitive-Behavioral Therapies with Lesbian, Gay, and Bisexual Clients.

[91] Safren S.A., Hollander G., Hart T.A., Heimberg R.G. (2001). Cognitive-behavioral therapy with lesbian, gay, and bisexual youth. Cogn. Behav. Pract..

[92] Stern C., Kleijnen J. (2020). Language bias in systematic reviews: You only get out what you put in. JBI Evid. Synth..

[93] Bajwa N.u.H., König C.J. (2019). How much is research in the top journals of industrial/organizational psychology dominated by authors from the US?. Scientometrics.

